# Influence of Cu on the Mechanical and Shape Memory Properties of TiNi Alloys

**DOI:** 10.3390/ma18102407

**Published:** 2025-05-21

**Authors:** Luzhou Dong, Weifang Mann, Bo He

**Affiliations:** 1 Research Center of High-Temperature Alloy Precision Forming, Shanghai University of Engineering Science, Shanghai 201620, China; dongluzhou1999@163.com; 2 School of Materials Science and Engineering, Shanghai University of Engineering Science, Shanghai 201620, China; 3 Shanghai Marine Equipment Research Institute, No. 10 Hengshan Rd., Shanghai 200031, China; qlsmwf@163.com

**Keywords:** TiNiCu SMAs, twin martensite, precipitation, hot rolling, ductility

## Abstract

The significant phase transformation hysteresis in TiNi alloys limits their performance. To address this, copper (Cu) was added as an alloying element to reduce hysteresis. This study synthesized three compositions of Ti_50_Ni_50−*x*_Cu*_x_* (x = 0, 5, 7 at.%) shape memory alloys (SMAs) via vacuum arc melting to optimize the Cu content. The alloys were homogenized through hot rolling to maintain stable mechanical and shape memory properties. The hot-rolled Ti_50_Ni_45_Cu_5_ alloy demonstrated excellent shape memory behavior, achieving 100% thermal recovery after one cycle at 4% and 6% strain and 99.2% recovery after six cycles at 4% strain. It also exhibited outstanding mechanical performance, with a tensile strength of 900 MPa and 40% elongation. Microscopic analysis using scanning electron microscopy (SEM) with energy-dispersive X-ray spectroscopy (EDS), electron backscatter diffraction (EBSD), and transmission electron microscopy (TEM) revealed that Cu preferentially segregates at grain boundaries, suppressing the formation of the Ti_2_(Ni,Cu) phase. This moderate segregation, combined with hot rolling, promotes the reprecipitation and uniform distribution of phases, reducing the likelihood of premature fracture caused by stress concentration during deformation. The moderate thickness and uniformly distributed martensite, as well as the Type II twins with strong deformation ability, significantly improved the shape memory properties of Ti_50_Ni_45_Cu_5_. This study provides valuable insights into the microscopic mechanisms influenced by Cu in TiNi alloys and proposes a novel strategy for controlling precipitate phases through adjustments in alloy composition and optimized processing conditions.

## 1. Introduction

TiNi alloys are renowned for their remarkable shape memory effect, hyperelasticity, corrosion resistance, and biocompatibility, making them suitable for biomedical applications [1]. However, these functional properties are highly sensitive to changes in composition, as even a 1 at.% increase in Nickel (Ni) content can lower the martensitic transformation temperature by approximately 100 °C, which limits the broader application of TiNi alloys [2].

To address these limitations, copper (Cu) was introduced as a partial replacement for Ni in binary TiNi alloys. This modification reduces the sensitivity of the martensitic transformation temperature to compositional changes [3], decreases transformation hysteresis, suppresses the R-phase transition, and enhances fatigue resistance and shape memory stability [4,5]. B. Strnadel et al. [6] reported that TiNiCu alloys demonstrate lower transformation strain and stress compared with TiNi alloys, resulting in reduced transformation hysteresis. However, as the number of cycles increases, the low critical resolved shear stress in these low-nickel alloys leads to higher residual plastic deformation. This behavior is due to the increased Cu content, which alters the lattice parameters [7] and lowers the strain required for the B2 to B19’ phase transformation. Consequently, the crystal structure becomes less stable, reducing the shear stress threshold and promoting the accumulation of residual plastic deformation within the martensitic phase. Therefore, optimizing the Cu content in TiNiCu alloys is essential to balance performance and functionality. Shiva et al. [8] demonstrated that Ti_50_Ni_45_Cu_5_ exhibits favorable phase transformation characteristics and improved microhardness, though its mechanical and functional properties were not extensively investigated.

The formation and evolution of precipitates in TiNi shape memory alloys (SMAs) are crucial factors that significantly impact their performance and functionality [5]. In Ti-rich TiNi alloys, high-temperature heat treatments often result in the formation of Ti_2_Ni precipitates. However, these precipitates can also cause premature fracture during the early stages of plastic deformation, leading to a pronounced reduction in ductility [9]. As a result, controlling Ti_2_Ni precipitation has become an important strategy for enhancing the properties and functionality of TiNi alloys. By precisely managing heat treatment processes, it is possible to optimize precipitate phase formation and improve the overall alloy performance [10]. H. Z. Lu et al. [11] demonstrated that complete solution treatment of additively manufactured TiNi alloys results in the formation of nanoscale, spherical Ti_2_Ni precipitates that are uniformly distributed within the grains, thereby enhancing both mechanical performance and shape memory behavior. In addition to heat treatment, the incorporation of Cu significantly influences the precipitate phase. C.J. de Araújo et al. reported that adding Cu into TiNi alloys results in discontinuous precipitates at grain boundaries, as Cu suppresses the formation of the Ni_4_Ti_3_ phase [12]. However, in equiatomic TiNi alloys where Cu replaces Ni, the precipitate phase commonly appears as Ti_2_(Ni,Cu), indicating that the mechanism described in [13] is inadequate to explain this phenomenon. Therefore, further investigation into Cu’s role in the formation of the Ti_2_Ni precipitate phase and its effects on the alloy’s properties and performance is crucial.

In addition, dislocations play a crucial role in the properties of TiNi shape memory alloys, and Liao et al. [14] in their study of Fe_64_(CoCrNi)_36_ in the hot-rolled state found that dislocation strengthening, grain boundary strengthening and hot rolling induced defects resulted in a significant improvement in yield strength. Due to the increase in yield stress, a significant phase transition induced plasticity (TRIP) effect supported the sustained uniform plastic deformation. Zhang et al. in their study of ordinary steel found that dislocation slip up to the dislocations across the interphases increased both strength and ductility [15]. In addition, dislocations can influence the driving force and resistance to martensitic phase transformations, thus affecting the shape memory properties.

In this study, Cu is partially substituted for Ni in equiatomic SMAs, followed by homogenization through hot rolling. The microscopic effects of Cu on TiNi-based SMAs are examined from various perspectives, including phase transformation temperatures, precipitate phase content, martensite morphology, and twinning behavior. In addition, this research provides new insights into controlling precipitate phases by combining alloy composition adjustments with process optimization.

## 2. Materials and Methods

### 2.1. Vacuum Arc Melting and Hot Rolling

Ti (99.9 wt.%), Ni (99.9 wt.%), and Cu (99.9 wt.%) of high purity were utilized to produce Ti_50_Ni_50−_*_x_*Cu*_x_* (*x* = 0, 5, 7 at.%) alloy ingots through vacuum arc melting. To achieve compositional uniformity, the ingots were inverted and remelted a total of eight times. A 10 g sample was extracted from the ingot. The Ni content was determined via gravimetric analysis (precipitation method), while O and H concentrations were measured using an oxygen/hydrogen/nitrogen analyzer (ONH5500, Beijing, China). All measurements were performed in triplicate, and the final results represent the average values. The Ti content was calculated by subtracting the sum of all other measured elements from 100 at.%. Detailed results are presented in Table 1. The ingots were then subjected to hot rolling at 850 °C, reducing their thickness to 1.5 mm, with each rolling pass reducing the thickness by 0.05 mm. Samples of the required dimensions were prepared through wire electrical discharge machining. The hot-rolled samples were designated as HR-TiNi, HR-TiNiCu_5_, and HR-TiNiCu_7_. For comparison, the as-cast samples were labeled As-TiNi, As-TiNiCu_5_, and As-TiNiCu_7_ to examine the improvements of hot rolling on performance.

### 2.2. Microstructure and Property Characterization

Phase transformation temperatures were determined using differential scanning calorimetry (DSC, Netzsch DSC 200 F3, Selb, Germany) in the temperature range of 193–413 K, under a nitrogen atmosphere, with heating and cooling rates of 10 °C/min. The sample dimensions were 3 mm × 2 mm × 1.5 mm, with a weight of 58 mg. Microstructural analysis was conducted on cubic samples measuring 10 mm × 10 mm × 1.5 mm. To enhance the visibility of the grain structure and precipitate distribution under optical microscopy (OM) and scanning electron microscopy (SEM), the samples were etched with a chemical solution of HF, HNO_3_, and H_2_O in a 1:4:5 volume ratio. Phase composition analysis was conducted on polished cubic samples using X-ray diffraction (XRD, D8 Advance, Karlsruhe, Germany) with Cu Kα radiation and SEM (TESCAN LYRA3 GUM, Brno, Czech Republic, acceleration voltage: 10–20 kV) equipped with energy-dispersive X-ray spectroscopy (EDS). Transmission electron microscopy (TEM) samples were prepared by sectioning rolled plates into cubes (10 mm × 10 mm × 0.5 mm) and further thinning them to a thickness of 80 μm using sandpaper. Circular discs with a diameter of Ø3 mm were punched from the thinned samples and electropolished at –30 °C in a solution of 4 vol.% perchloric acid and 96 vol.% ethanol using the Struers TenuPol-5 system (Ballerup, Denmark). Subsequent ion beam thinning was performed with a Gatan 691 system (Pleasanton, CA, USA). High-resolution TEM (FEI Tecnai F30, Hillsboro, OR, USA) imaging was conducted at 200 kV using the transient electromagnetic method.

Samples for electron backscatter diffraction (EBSD, EDAX Hikari Plus, Mahwah, NJ, USA) analysis were prepared with dimensions 10 mm × 10 mm × 1.5 mm. The surfaces were ground sequentially using 220, 600, 1000, 1500, 2000, and 5000 grit sandpapers, followed by polishing with a 0.05 μm solution to achieve a mirror finish. Electrochemical polishing was performed in a solution of 30 vol.% nitric acid and 70 vol.% methanol at −20 °C for 30 s, using an applied voltage of 20 V and a current of 0.8 A. The test was performed in step of 0.7~1.4 μm.

**Figure 1:**
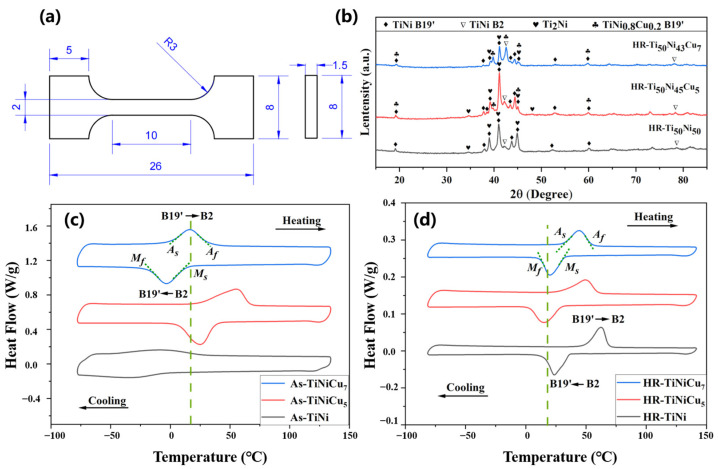
(**a**) Dimensions of the tensile samples; (**b**) XRD patterns of the hot-rolled alloys; (**c**) DSC curves for the as-cast; (**d**) DSC curves for the hot-rolled alloys.

Dog-bone-shaped samples, shown in Figure 1a, were prepared for performance and functional testing. These samples, cut along the rolling direction, had a cross-sectional width of 2 mm, a thickness of 1.5 mm, and a length of 10 mm. Tensile and shape memory effect (SME) tests were conducted at room temperature (20 °C) using a Shimadzu AGS-X testing machine, with tensile strain measured by an extensometer (ESA-CU200, Hatfield, PA, USA). SME testing involved tensile loading at 0.1 mm/min to strains of 4% and 6%, followed by unloading at the same rate. After several cycles, the samples were heated to a temperature of 30 °C above the austenite finish temperature (*A_f_*) and held for 5 min to assess recoverable strain.

## 3. Results and Discussion

### 3.1. Impact of Cu Content on Phase Transformation Behavior

Figure 1c,d present the DSC curves for the as-cast and hot-rolled Ti_50_Ni_50-x_Cuₓ (x = 0, 5, 7 at.%) alloys, with the green dashed line indicating room temperature. All the samples displayed the B2 (cubic austenite) ⇆ B19’ (monoclinic manensite) single-step phase transformation. Nam et al. investigated the effect of Cu on the phase transformation of TiNi alloys and found that the B2 ⇆ B19 (rhombohedral martensite) ⇆ B19’ two-step martensitic phase transformation occurs at Cu contents above 7 at.% [16]. However, this transformation did not occur in the HR-TiNiCu_7_ alloy. From the DSC curves, phase transformation temperatures (*M_s_*, *M_f_*, *A_s_*, and *A_f_*), transformation enthalpies (∆*H_MA_* and ∆*H_AM_*), and transformation hysteresis (*D_TH_*) were calculated, as outlined in Table 2. The comparison of phase transformation temperatures before and after hot rolling reveals that TiNi alloys experience temperature fluctuations of several tens of degrees Celsius. This is mainly attributed to the inhomogeneous chemical composition of the as-cast samples. As indicated in Table 1, the phase transformation temperatures of TiNi alloys are highly sensitive to compositional variations. After homogenization via hot rolling, the phase transformation temperatures of TiNi exhibit significant fluctuations. However, the addition of copper markedly reduces the issue of compositional inhomogeneity, leading to a corresponding decrease in the fluctuation of phase transformation temperatures. Notably, TiNiCu_5_ exhibited the greatest thermal stability, with the martensitic and austenitic start temperatures varying by only about 7 °C after hot rolling. This demonstrates that incorporating a small amount of copper into TiNi alloys can enhance the homogeneity of the alloy composition during sample preparation, thereby improving the stability of the phase transformation. Furthermore, hot rolling led to a noticeable decrease in transformation enthalpy, likely due to the introduction of dislocations, leading to the formation of stable martensite. Despite this, in both its as-cast and hot-rolled states, TiNiCu_5_ consistently showed the highest transformation enthalpy among the alloys, suggesting that an optimal Cu content may promote a more efficient phase transformation process.

The measured transformation hysteresis of the TiNi was 33.5 °C. With the addition of 5 at.% Cu, the hysteresis (*D_TH_* = *A_f_* − *M_s_*) decreased to 29.3 °C and further reduced to 19.0 °C with 7 at.% Cu. In the hot-rolled samples, HR-TiNi exhibited a transformation hysteresis of 32.1 °C, which dropped to 30.0 °C for HR-TiNiCu_5_ and 22.3 °C for HR-TiNiCu_7_. These results demonstrate that transformation hysteresis is inversely related to Cu content, consistently decreasing as Cu content increases. This trend is in agreement with the findings of Tae Hyun Nam et al. [5].

### 3.2. Effect of Cu Addition on the Formation and Evolution of Precipitate Phases

Figure 1b presents the XRD patterns of TiNi and TiNiCu alloys following hot rolling. The HR-TiNi alloy predominantly contains the TiNi B19’ martensite phase, the B2 austenite phase, and the Ti_2_Ni precipitate phase. With the introduction of Cu, the Ti_2_Ni phase transforms into Ti_2_(Ni,Cu), and the peaks corresponding to the B2 and B19’ phases shift to the right. This shift is likely due to Cu’s influence on the lattice parameters. In addition, a TiNi_0.8_Cu_0.2_ B19’ phase, containing up to 10 at.% Cu was detected alongside the TiNi B19’ phase.

**Figure 2:**
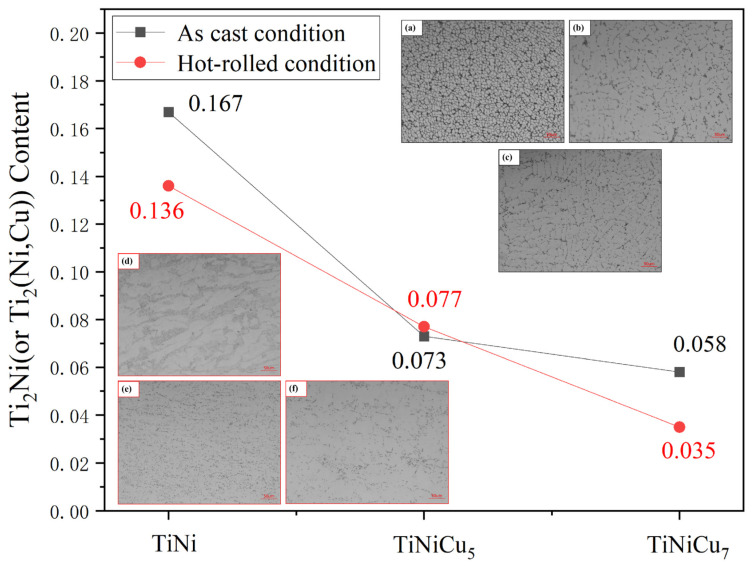
Trends in Ti_2_Ni content and OM images: (**a**) As-TiNi; (**b**) As-TiNiCu_5_; (**c**) As-TiNiCu_7_; (**d**) HR-TiNi; (**e**) HR-TiNiCu_5_; (**f**) HR-TiNiCu_7_.

Optical microscopy, performed after etching both as-cast and hot-rolled samples, revealed continuous precipitation along the grain boundaries in the As-TiNi alloy. By contrast, the As-TiNiCu_5_ and As-TiNiCu_7_ alloys exhibited discontinuous grain boundary precipitates, as shown in Figure 2a–c. Furthermore, the number of precipitates decreased with increasing Cu content, as indicated by the trend line in Figure 2. This trend was consistent in the hot-rolled samples, suggesting that the addition of Cu inhibits precipitation formation. Preliminary XRD analysis identified the precipitate phase in the TiNi alloy as Ti_2_Ni. Figure 2d–f show that after hot rolling, the Ti_2_Ni precipitates in HR-TiNi, although irregularly shaped, continued accumulating at grain boundaries. In contrast, the precipitates in the HR-TiNiCu alloys, especially HR-TiNiCu_5_, were more dispersed and exhibited a more uniform distribution.

**Figure 3:**
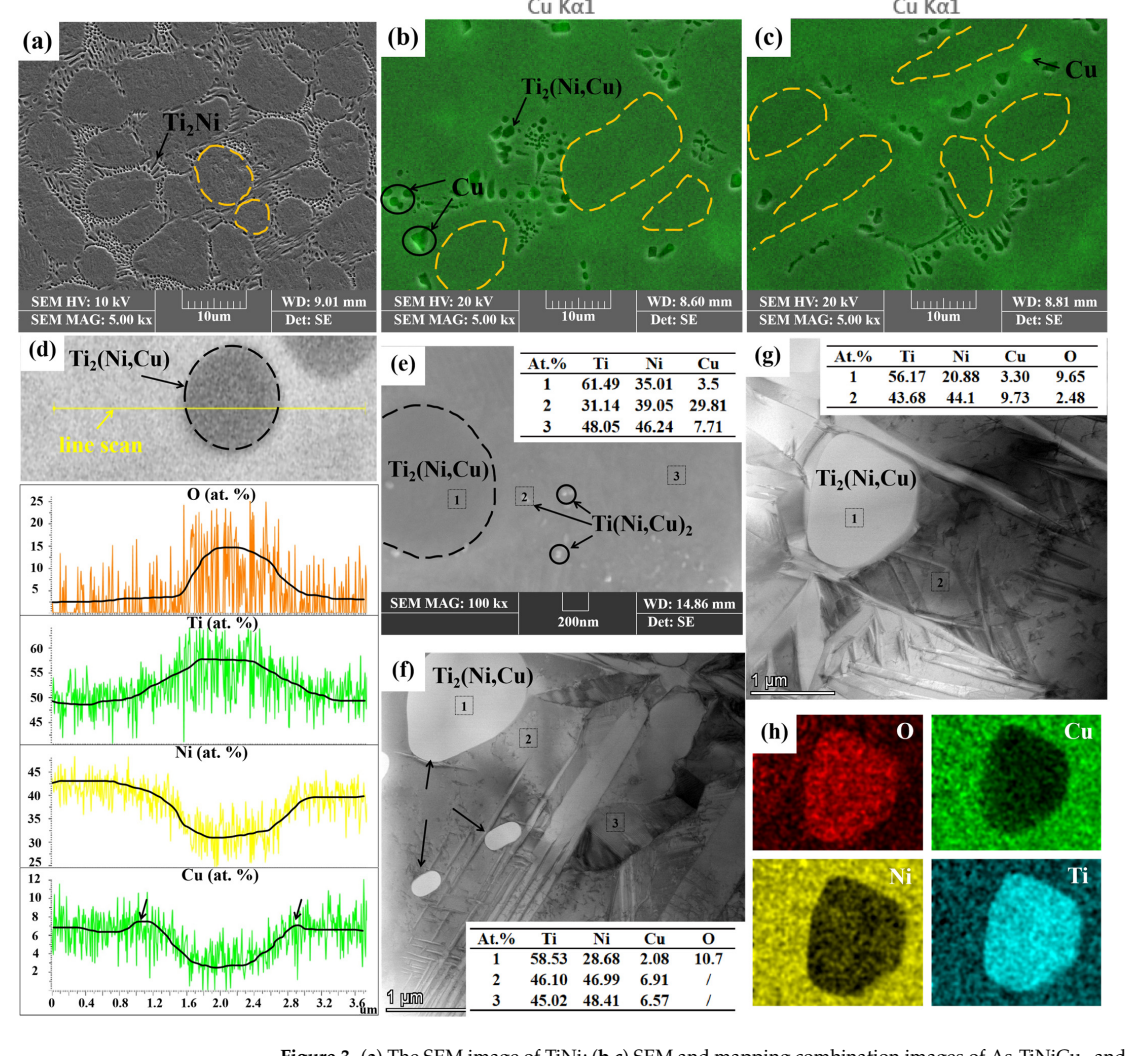
(**a**) The SEM image of TiNi; (**b**,**c**) SEM and mapping combination images of As-TiNiCu_5_ and As-TiNiCu_7_; (**d**) Ti_2_(Ni,Cu) line scan of As-TiNiCu; (**e**) SEM image of HR-TiNiCu_5_; (**f**,**g**) HR-TiNiCu_5_ and HR-TiNiCu_7_ high angle annular dark field images (HAADF) in transmission electron microscopy; (**h**) mapping image corresponding to (**g**).

**Figure 4:**
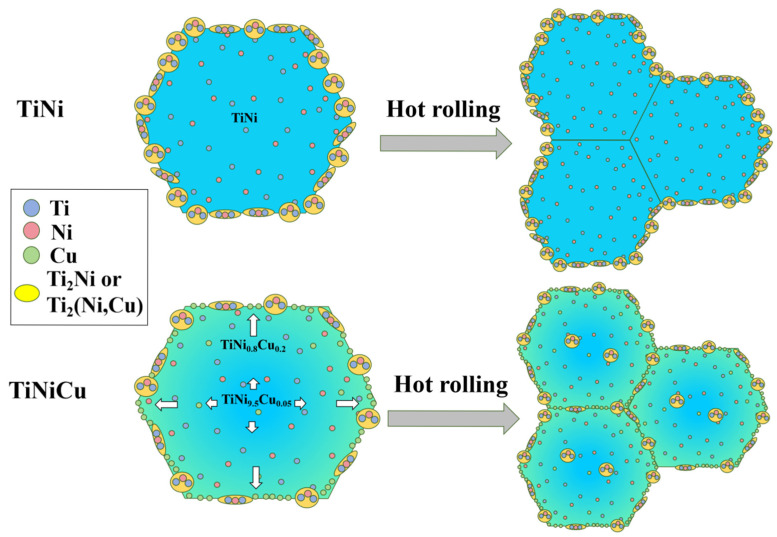
Influence of hot rolling and copper elements on the precipitation phases of TiNi and TiNiCu alloys and mechanisms of evolution.

Figure 3a,c show SEM images of the etched as-cast samples. Compared with the as-cast TiNi, the distribution of precipitates at the grain boundaries becomes noticeably discontinuous with the addition of Cu. To better understand the effects of Cu on the microstructure of TiNi alloys, an EDS analysis was performed. The line scan in Figure 3d reveals that the Ti content is roughly twice that of the combined Ni and Cu contents, confirming the precipitate as Ti_2_(Ni,Cu). This phase forms by replacing some Ni with Cu in the Ti_2_Ni structure while retaining the same crystal structure as Ti_2_Ni [17]. Notably elevated oxygen content was observed in the Ti_2_Ni/Ti_2_(Ni,Cu) precipitates (Figure 3d). This phenomenon can be attributed to the strong affinity of the Ti_2_Ni phase to react with oxygen, leading to the formation of Ti_4_Ni_2_O_x_ phases, as reported in prior studies [18,19]. Both Ti_2_Ni and Ti_4_Ni_2_O_x_ belong to the cubic crystal system. The higher oxygen concentration quantified in Table 1 aligns with this structural characteristic. Notably, the Cu content around the precipitates was higher than in the matrix (5 at.%), peaking at the precipitate edges, as indicated by the arrow in Figure 3d. A map scan of the As-TiNiCu samples was conducted to visualize Cu distribution, and the Cu maps were superimposed onto the SEM images, resulting in Figure 3b,c. These images show that Cu tends to accumulate at grain boundaries due to the smaller size of Cu atoms and their lower formation energy at these boundaries [20]. The peak marked by the arrow in Figure 3d likely results from the precipitates hindering the diffusion of Cu diffusion along the grain boundary, leading to local accumulation. In turn, the precipitation of Ti_2_(Ni,Cu) is hindered by the diffusive movement of Cu at grain boundaries, which results in a decrease in the content of precipitated phases and a discontinuous distribution of precipitated phases after the addition of Cu to TiNi alloys, as demonstrated in Figure 4 (mechanism diagram before hot rolling).

To eliminate the possibility that the observed phenomena were due to compositional inhomogeneity in the as-cast samples, a similar elemental distribution analysis was conducted on the hot-rolled specimens. Figure 3f–h present the results of point and map scans in high angle annular dark field (HAADF) mode for HR-TiNiCu_5_ and HR-TiNiCu_7_. These scans indicate that the Cu content within the precipitates is lower than in the matrix (5 at.%), while the Cu concentration surrounding the precipitates is elevated, exhibiting a gradient of increasing Cu levels approaching the precipitates. This observation is consistent with the findings from the as-cast samples, indicating that Cu tends to segregate at the grain boundaries in TiNiCu alloys [21]. This is also consistent with the observed result of TiNi_0.8_Cu_0.2_ in XRD. At the grain boundaries, the presence of enriched Cu results in the replacement of Ni in the lattice, thereby causing the alloy to transform from the TiNi B19’ phase to TiNi_0.8_Cu_0.2_ B19’. However, no clear delineation between TiNi B19’ and TiNi_0.8_Cu_0.2_ B19’ was observed under SEM. This is attributable to the increment in Cu elemental content from the matrix to the grain boundaries.

Kim et al. found that precipitation phases in the TiNi_0.8_Cu_0.2_ alloy will initially solidify into the matrix under the action of the hot rolling temperature, thereby forming a supersaturated solid solution [22]. Thereafter, the precipitated phase is re-precipitated and more evenly distributed. This is one of the factors contributing to the uniform and diffuse distribution of precipitated phases following the hot rolling of TiNiCu alloys. Furthermore, the tendency of Cu atoms to polarize at grain boundaries, which results in a reduction in grain boundary energy, has been demonstrated to promote the rate of grain boundary migration during the hot rolling process. The migration of grain boundaries provides a driving force for the movement of precipitation phases, resulting in a more diffuse distribution of TiNiCu alloy precipitation phases. In contrast, TiNi alloys without Cu addition exhibit localized precipitate distribution exclusively at immobile grain boundaries during hot rolling, as schematically illustrated in Figure 4 (microstructural evolution mechanism). The grain morphology of the alloys after hot rolling in this diagram is mentioned in Section 3.3.

In the HR-TiNiCu_5_ alloy, a white nanoscale precipitate (20–40 nm) was identified as shown in Figure 3e. Point scan analysis reveals that the Ni and Cu contents are approximately double that of Ti, indicating the precipitate is likely Ti(Ni,Cu)_2_. The formation of this precipitated phase is attributed to the Cu segregation, which reduces the formal energy of the precipitated phase and thus promote the formation of the nanoscale precipitated phase [23]. At the same time, enrichment of Cu at grain boundaries reduce the local equilibrium free energy between the grain boundaries and the matrix, stabilize the grain boundaries, and provide a favorable environment for the precipitation of nanoscale Ti(Ni,Cu)_2_ precipitated phase with high Cu [24,25]. The presence of the Ti(Ni,Cu)_2_ phase is known to result in enhanced elongation properties in TiNiCu alloys [26].

### 3.3. Effect of Cu on the Organization of Grains in Both Martensite and Austenite

**Figure 5:**
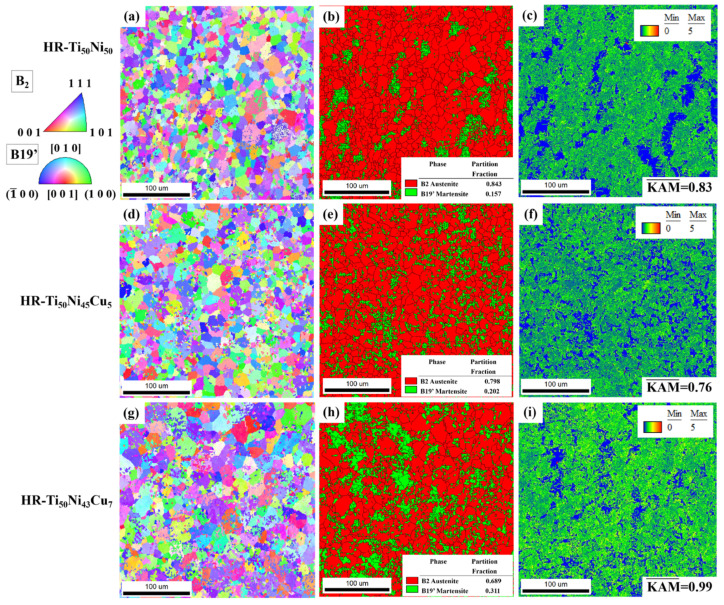
EBSD results of HR-NiTi and HR-TiNiCu samples along rolling direction: (**a**,**d**,**g**) inverse pole figure (IPF) maps; (**b**,**e**,**h**) phase maps for B2 and B19’; (**c**,**f**,**i**) KAM maps.

The yellow dashed circle in Figure 3 indicates that the incorporation of Cu results in a significant increase in grain size. To further investigate this phenomenon, an EBSD analysis was conducted. Figure 5 presents the EBSD grain morphology, along with phase maps for B19’ and B2, as well as kernel average misorientation maps. As demonstrated in Figure 5a,d,g, it is evident that the three alloys exhibit no discernible orientation relationship subsequent to hot rolling, and all of them are approximately equiaxial crystals. As illustrated in Figure 5b,e,h, the martensite content was 15.7% for HR-TiNi, 20.2% for HR-TiNiCu_5_, and 31.1% for HR-TiNiCu_7_. The specimens underwent martensitic transformation at −20 °C during preparation, followed by a return to room temperature. Figure 1d confirms that the austenitic transformation temperatures for all three samples exceed room temperature, indicating that no austenitic transformation occurred in any of the samples. This observation substantiates the conclusion that all specimens remained in the low-temperature phase during the EBSD analysis. These findings suggest that an increase in Cu content correlates with a higher martensite content, implying that elevated Cu concentrations may facilitate the martensitic transformation. Moreover, the crystal structures distribution graph (see Figure 5c,f,i) demonstrates that the kernel average misorientation (KAM) of the three alloys post-hot rolling are also disparate. HR-TiNiCu_5_ was found to have the lowest average KAM ($\bar{KAM}$) at 0.76, followed by HR-TiNi at 0.83 and HR-TiNiCu_7_ as the highest at 0.99.

**Figure 6:**
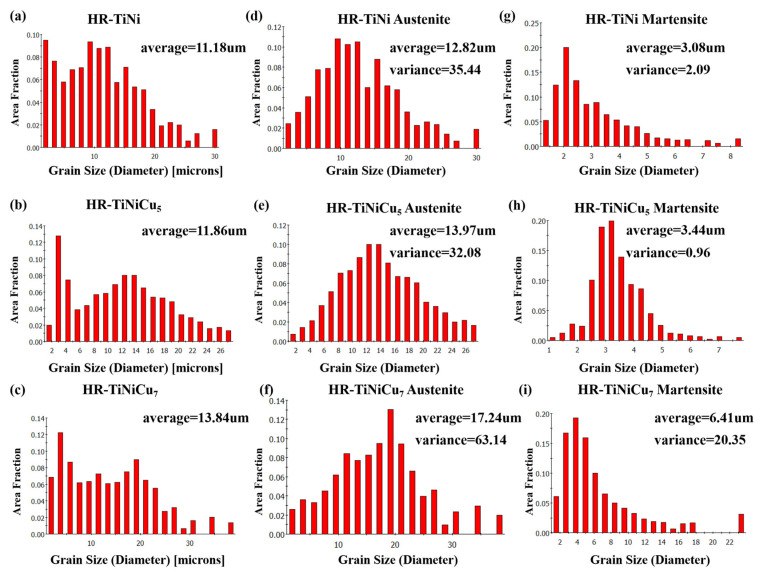
Statistical histograms of overall grain size, B19’ and B2 phase grain size, and mean and variance are shown in the left, middle, and right columns, respectively: (**a**,**d**,**g**) HR-TiNi; (**b**,**e**,**h**) HR-TiNiCu_5_; (**c**,**f**,**i**) HR-TiNiCu_7_.

It is evident that the grain size increases in proportion to the increase in Cu content. Quantitative analyses provide further evidence to support this, with the average grain size of HR-TiNi recorded as 11.18 µm, and with the addition of Cu, the average grain size of HR-TiNiCu_5_ increasing to 11.86 µm, and that of HR-TiNiCu_7_ increasing further to 13.84 µm, as shown in Figure 6a–c. This grain size increase can be attributed to the presence of Cu, which promotes grain boundary migration and accelerates the rate of grain growth [27]. Furthermore, Figure 5b,e,h demonstrate a marked difference in size and spatial distribution between martensite and austenite phases. Consequently, the martensite and austenite grains were analyzed separately, as illustrated in Figure 6d–i. It was determined that HR-TiNiCu_5_ exhibits the most uniform grain size of both phases, in addition to the highly uniform spatial distribution of both phases. The variance in the austenite grain size was determined to be 32.08, while the martensite grain size exhibited a significantly lower variance of 0.96.

**Figure 7:**
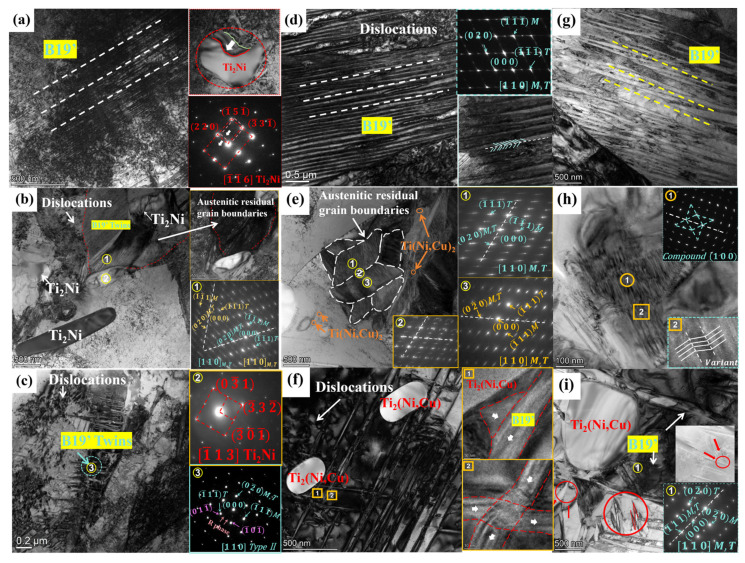
TEM microstructure of dislocations, B19’ martensite, and Ti_2_Ni precipitates: (**a**–**c**) HR-TiNi; (**d**–**f**) HR-TiNiCu_5_; (**g**–**i**) HR-TiNiCu_7_.

Figure 7 presents TEM images of the three alloys, each displaying both the B2 austenite and B19’ martensite phases, along with a high density of dislocations. As illustrated in Figure 7a,d,g, the bright and dark field images of the three alloys under TEM reveal the presence of different martensitic phases. The enlarged dark field image of HR-TiNi demonstrates that the martensite is characterized by significant slimness, with an average thickness of approximately 10 nm. Unlike HR-TiNi, HR-TiNiCu_5_ displays a considerable number of martensitic lath groups, as shown in Figure 7d. The martensitic group exhibits the presence of two variants, with different orientations growing along the same direction. These variants are uniform in size and have a thickness of approximately 30 nm. In addition, Figure 7f depicts slender martensite variants with varying growth orientations that intersect around the Ti_2_(Ni,Cu) precipitates. The high-resolution analysis further illustrates that the martensite variants intertwine at these intersection points, forming kinks before continuing to grow in a coordinated manner, resulting in an overall ordered growth direction. Figure 7g illustrates that HR-TiNiCu_7_ displays martensitic lath groups akin to those found in HR-TiNiCu_5_; however, these lath groups exhibit uneven thickness, averaging approximately 50 nm. In addition, Figure 7i shows intersecting martensite variants (highlighted by the red circle) that form a “Z”-shaped configuration, which corresponds to the martensitic Type I twins.

The TEM samples were prepared by subjecting them to cryogenic treatment, followed by an elevation to room temperature. It can be concluded from the phase transition temperatures that no austenitic phase transition occurred during this process. However, residual austenite grain boundaries were observed in both HR-TiNi and HR-TiNiCu_5_ by projection electron microscopy, as illustrated in Figure 7b,e. This phenomenon was not observed in HR-TiNiCu_7_. The residual austenite undergoes martensitic phase transformation under stress, which can provide additional deformation capacity and thus improve the stability and reversibility of the shape memory effect [28,29].

### 3.4. Tensile Properties and Shape Memory Effect

**Figure 8:**
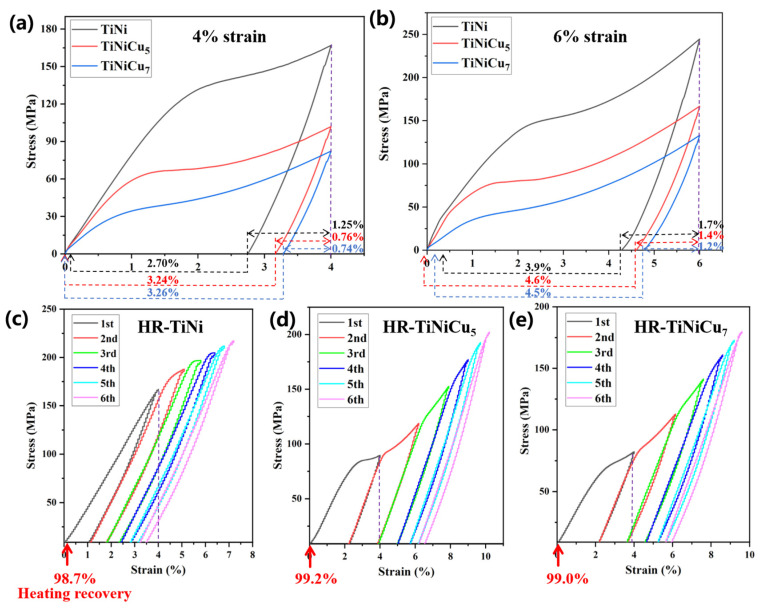
Tensile loading–unloading curves. (**a**) One-cycle tension-heating at 4% maximum strain; (**b**) one-cycle tension-heating at 6% maximum strain; (**c**–**e**) six-cycle tension-heating at 4% strain. The arrows at the horizontal coordinates are for the case of heating recovery, labeled with the recovery strain and recovery rate.

To investigate the shape memory properties of the alloys, tensile cycling tests were conducted at strain levels of 4% and 6%, as illustrated in Figure 8a,b. After each cycling session, the samples were heated to A_f_ + 30 °C and held at this temperature for five minutes. At the 4% strain level, HR-TiNiCu_5_ and HR-TiNiCu_7_ achieved complete shape recovery, while HR-TiNi showed a recovery rate of 98.8%. The HR-TiNi alloy demonstrated a recovered strain of 1.25% upon unloading and 2.7% after being heated. By contrast, HR-TiNiCu_5_ and HR-TiNiCu_7_ exhibited a lower unloaded recovery strain of about 0.75%, yet they recorded a higher thermal recovery strain of over 3.25%. For a strain of 6%, HR-TiNiCu_5_ achieved a recovery of more than 4.6% upon heating, resulting in a total recovery rate of 100%. Conversely, HR-TiNiCu_7_ and HR-TiNi had a recovery rate of 95% and 93.3%, respectively. During the following six tensile cycles at a 4% strain, HR-TiNiCu_5_ maintained the highest thermal recovery rate at 99.2%, as illustrated in Figure 8c–e. Overall, HR-TiNiCu_5_ demonstrated the most remarkable shape memory capabilities. The shape memory effect test was conducted at room temperature. Future research will assess different applications—specific temperatures to meet real-world thermal requirements. Costanza et al. [30] tested commercial TiNi sheets at various fixed temperatures and found that the maximum load during the shape memory process increases with temperature.

**Figure 9:**
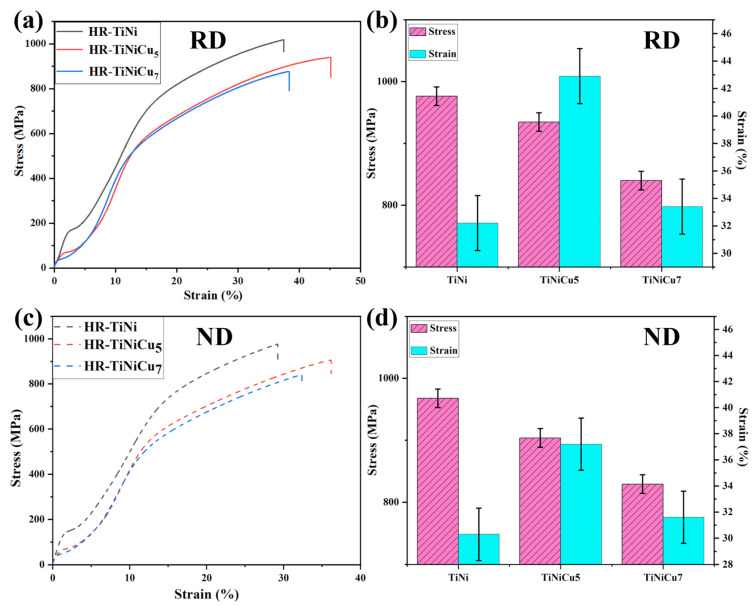
Tensile stress–strain curves of hot-rolled samples: (**a**) rolling direction (RD); (**c**) normal direction (ND). The ultimate strengths and strains of the three alloys are also compared: (**b**) RD; (**d**) ND.

Owing to the inhomogeneous composition of the cast samples, their mechanical properties exhibit significant variability, and their characteristic values are provided exclusively in Table 3. A clear enhancement in the properties and stability of the three alloys is evident following hot rolling, as evidenced by the data. Figure 9 presents the tensile stress–strain curves of the hot-rolled samples. Figure 9a,c display the stress–strain curves along the rolling direction (RD) and normal direction (ND), respectively. Figure 9b,d provide a comparative visualization of the ultimate tensile strength (UTS) and fracture strain values across the tested conditions. It was determined that the strength of the alloy is inversely proportional to the Cu content. The initial platform exhibits a similar pattern, as outlined in Table 3. In contrast, the elongation follows a distinct trend: HR-TiNiCu_5_ demonstrates the highest ductility with values of 44.9 ± 2% (RD) and 38.2 ± 2% (ND), followed by HR-TiNiCu_7_ (32.4 ± 2% (RD) and 31.6 ± 2% (ND)) and HR-TiNi (29.2 ± 2% (RD) and 30.3 ± 2% (ND)). A comparative analysis of TiNiCu alloy and TiNi alloy reveals that the former exhibits significantly enhanced elongation after hot rolling. This enhancement is attributed to the optimal Cu content, which ensures superior performance.

**Figure 10:**
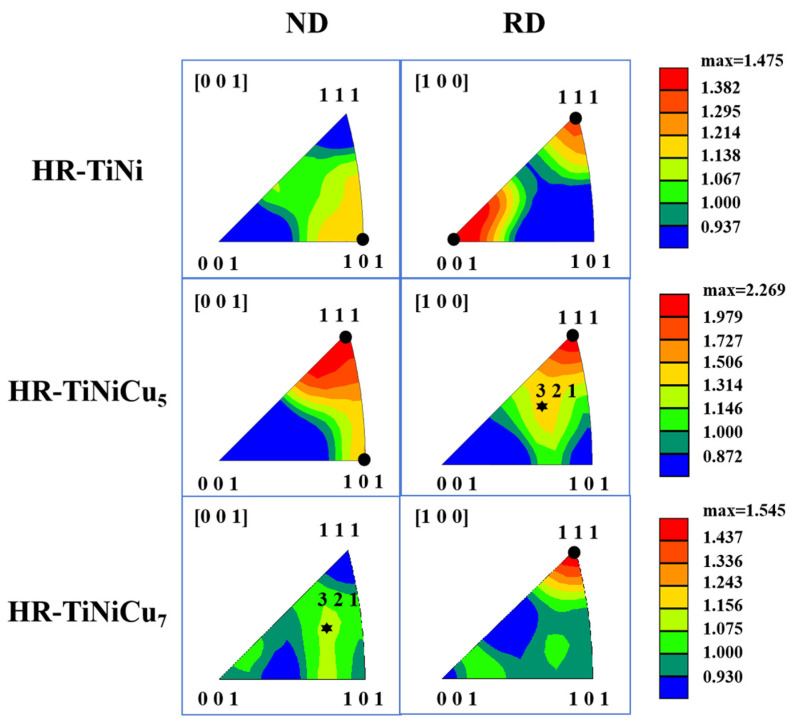
Inverse pole figures (IPFs) of the three alloys after hot rolling.

Figure 10 presents the inverse pole figures (IPFs) of the three alloys. In HR-TiNi, a strong {111}//RD (rolling direction) texture and a {001}//RD texture are observed, along with a weaker {101}//ND (normal direction) texture. HR-TiNiCu_5_ exhibits dominant {111}//RD and {111}//ND textures, coupled with a minor {321}//RD component. For HR-TiNiCu_7_, the microstructure is characterized by a predominant {111}//RD texture and a faint {321}//ND texture. During tensile testing along the rolling direction (RD), the dominance of the {001}//RD texture in HR-TiNi significantly enhances tensile strength, albeit at the expense of plasticity. In contrast, the {111}//RD texture prevailing in HR-TiNiCu_5_ and HR-TiNiCu_7_ promotes dislocation slip during deformation, thereby improving plasticity while exerting limited influence on tensile strength. Notably, HR-TiNiCu_5_, which possesses the strongest {111}//RD texture, achieves the highest plasticity among the three alloys. In the normal direction (ND), the {101}//ND texture in HR-TiNi contributes to elevated tensile strength but does not favor plasticity. Conversely, HR-TiNiCu_5_ demonstrates enhanced plasticity due to its dominant {111}//ND texture. Although HR-TiNiCu_7_ lacks a pronounced texture and exhibits lower plasticity compared to HR-TiNiCu_5_, it still surpasses HR-TiNi in ductility.

## 4. Discussion

TiNiCu alloys with varying Cu contents exhibit distinct build-up of Cu content at grain boundaries and varied mis-slip mechanisms [31]. These interlocking behaviors give rise to differences in the twin-related martensitic plates that are formed. The martensite of HR-TiNi is predominantly present in the form of elongated flat forms. In contrast, the presence of short and different orientated type II twin-related martensite plates is indicative of dislocation plugging, as illustrated in Figure 7c (yellow circle 3). While the martensitic plates with type II twins exhibit a satisfactory deformation capacity, their size is inadequate and their resistance to deformation is constrained [32]. Furthermore, the TiNi alloy exhibits residual austenite grain boundaries, accompanied by the presence of very fine martensite bar bundles, as evidenced in Figure 7b. This martensitic block presence of multiple twinned crystals [33]. In yellow circle 1, a <110> Type II twin intersects with a (100) compound twin, demonstrating mirror symmetry with respect to the (100) plane. A 60° counterclockwise rotation about the (100) axis reveals another <110> Type II twin, symmetrically aligned with the (100) twin plane. The martensitic lath groups in HR-TiNiCu_5_ display <110> Type II twins. Residual austenite provided additional deformation capacity by undergoing stress-induced martensitic phase transformation during subsequent deformation. However, the high density of dislocations present in HR-TiNi hinders this process, leading to the formation of stable martensite with limited reversibility. In the HR-TiNiCu_7_ specimen, the presence of (100) composite twins and a substantial number of “Z”-shaped lamellae of type I martensite was observed, as illustrated in Figure 7h,i yellow circle 1. It is well established that martensitic plates with type I twins require higher stress during deformation for twinning to take place than those with type II twins, which corresponds to the high dislocation density of HR-TiNiCu_7_. This twin crystal exhibits a comparatively low deformation capacity and is susceptible to microcracks during deformation, as illustrated by the red circle in Figure 7i. These cracks may extend further and lead to a brittle fracture of the material. Furthermore, the absence of residual austenite grain boundaries in HR-TiNiCu_7_ was observed.

The HR-TiNiCu_5_ alloy demonstrates exceptional micro-morphology, attributable to the optimal addition of Cu content. The martensite of HR-TiNiCu_5_ is primarily characterized by martensite type II twins, with a uniform thickness of the martensite bar bundle. The self-regulating growth ability of the type II twin crystal is well demonstrated in the martensite, as evidenced by the presence of twisted and entangled places. This twin crystal requires low stress during deformation, resulting in relatively strong deformation ability. Furthermore, the HR-TiNiCu_5_ specimen exhibited the presence of austenitic residual grain boundaries, in addition to the internal presence of martensitic bar bundles displaying varied orientations, as illustrated in Figure 7e, yellow circles 1–3. Electron diffraction spot analysis confirms the same twinning structure in HR-TiNi, where <110> Type II twins intersect with (100) compound twins, exhibiting mirror symmetry relative to the (100) plane. The uniform and appropriate dislocation density of HR-TiNiCu_5_ does not adversely affect the stress-induced martensitic phase transformation dominated by this residual austenite, as well as the heating recovery phase.

**Figure 11:**
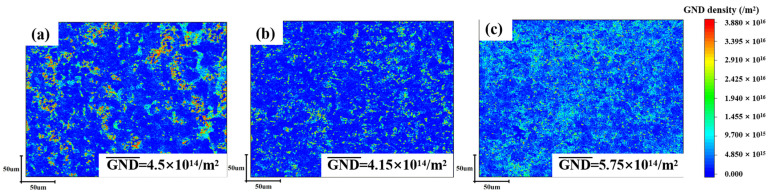
Dislocation distribution images: (**a**) HR-TiNi; (**b**) HR-TiNiCu_5_; (**c**) HR-TiNiCu_7_.

**Figure 12:**
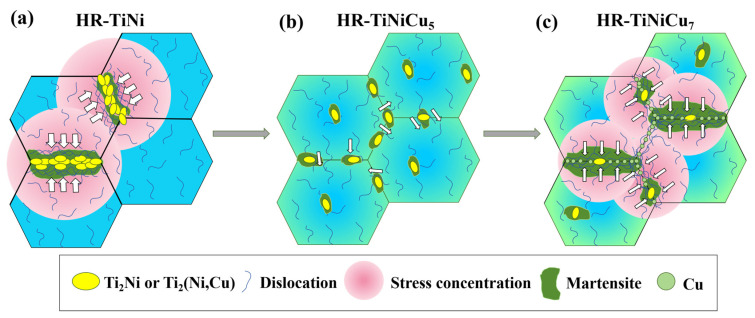
Mechanisms of the effect of precipitation phases/Cu elements on dislocation slip in three alloys, and stress concentration: (**a**) HR-TiNi; (**b**) HR-TiNiCu_5_; (**c**) HR-TiNiCu_7_. The white arrows indicate slip of dislocations.

As illustrated in Figure 11, the distribution of high-density dislocations varies significantly among the three alloys. In the case of TiNi alloy, high-density dislocations are distributed in the precipitation phase region, as illustrated in Figure 11a. As outlined in Section 3.2, the Ti_2_Ni precipitated phase of HR-TiNi is characterized by significant aggregation at grain boundaries. As demonstrated in Figure 12a, the dislocation slip that occurs during the hot rolling of the alloy is subsequently accumulated at this point by precipitation. This leads to stress concentration, consequently resulting in the unevenness of the Ti_2_Ni-induced martensitic phase transformation. The dislocation distribution depicted in Figure 11a, along with the misalignment of the precipitated phase resulting from dislocation slip, as illustrated in Figure 7a, provides substantial evidence that substantiates this perspective. The clogging of dislocation slip produces residual martensite, which demonstrated to significantly reduce the reversibility of shape memory properties. The interaction of stress concentration and dislocation blockage results in stress inhomogeneity during stress deformation of the alloy. This, in conjunction with the local distribution of brittle Ti_2_Ni, leads to an increased generation of crack sources, which results in a significant loss of properties [34,35].

The HR-TiNiCu_5_ precipitated phase is observed to be diffusely distributed following hot rolling. In contrast, HR-TiNiCu_7_ exhibits a different distribution of the precipitated phase. This is attributable to the fact that the higher Cu element exerts a dominant influence, thereby enhancing the inhibition of the precipitated phase at the grain boundaries. The amount of precipitated phase is insufficient to resist dislocation slip. When dislocations slip to the grain boundaries, the high content of Cu is firmly pinned at these boundaries, forming a high density of plugging and stress concentration, as illustrated in Figure 11c and Figure 12c. It is evident that a significant degree of austenite transformation into the martensitic phase occurs, resulting in the retention of excessive residual martensitic phases by high-density dislocations. This phenomenon is detrimental to performance and functionality.

The uniform distribution of Ti_2_(Ni,Cu) in HR-TiNiCu_5_ shown to be an effective method of resisting dislocation slip without generating local stresses. Furthermore, the appropriate Cu content is insufficient to produce dislocation plugging, as demonstrated in Figure 11b and Figure 12b. This, in turn, leads to a uniform distribution of martensitic nucleation locations during the phase transformation process (see Figure 5e). The favorable environment results in uniform grain size growth and uniform thickness of martensite plates of the alloy, as shown in Figure 7d,f. This results in a high synchronization of stress-induced martensitic phase transformations as well as martensitic reorientation under externally applied forces, leading to excellent shape memory properties [36].

## 5. Conclusions

This study systematically investigates the role of Cu in tailoring microstructural evolution and precipitate behavior (e.g., Ti_2_Ni, Ti_2_(Ni,Cu)) in TiNi-based alloys. By optimizing Cu content (5–7 at.%) and hot rolling parameters, we demonstrate precise control over precipitate morphology and distribution, leading to enhanced shape memory properties and mechanical performance. These findings provide a robust framework for designing high-performance TiNiCu alloys in precision-driven applications, such as biomedical stents and aerospace actuators. The key findings are outlined as follows:

(1) The HR-TiNiCu_5_ shape memory alloy (SMA) demonstrates outstanding shape memory performance, achieving a recovery rate of 99.2% upon heating after six tensile cycles at 4% strain. Furthermore, it exhibits exceptional tensile properties, with an elongation of 44.9 ± 2% and an ultimate tensile strength of 924.6 MPa ± 20 MPa, outperforming most previously reported TiNiCu SMAs under comparable processing conditions.
(2) This study posits that by adjusting the Cu content and utilizing the hot rolling process, the Ti_2_(Ni,Cu) phase can be modulated to optimize the dislocation slip mechanism, thereby improving the properties and functionality of TiNiCu alloys. This method provides novel insights into the modulation of precipitation phases.
(3) In this paper, the effect of the Cu element on the precipitation phase, phase transition temperature, grain size, and martensitic twinning of the TiNi alloy is elaborated. The impact of Cu on microstructure evolution is substantiated. Furthermore, this study proposes an optimized Cu ratio for TiNiCu alloys, thus providing a valuable reference point for industrial applications.

Future work will focus on additive manufacturing (AM) of TiNiCu alloys to address complex geometry requirements. Combining AM with tailored heat treatments (e.g., gradient aging, laser annealing) is proposed to further optimize phase transformation behavior and mechanical anisotropy.

## Figures and Tables

**Table 1:** Comparison of nominal and actual chemical compositions of the alloy obtained through melting.

Scheme		Ti (at.%)	Ni (at.%)	Cu (at.%)	O (at.%)	H (at.%)
Ti_50_Ni_50_	Nominal composition	50.00	50.00	/	/	/
	Actual component	49.15	50.48	/	0.33	0.04
Ti_50_Ni_45_Cu_5_	Nominal composition	50.00	45.00	5.00	/	/
	Actual component	50.03	44.85	4.91	0.33	0.04
Ti_50_Ni_43_Cu_7_	Nominal composition	50.00	43.00	7.00	/	/
	Actual component	50.03	42.81	6.95	0.33	0.03

**Table 2:** Values of *M_s_*, *M_f_*, *A_s_*, *A_f_*, ∆*H_MA_*, ∆*H_AM_*, and *D_TH_* obtained from the DSC curves of both as-cast and hot-rolled samples.

Sample	*A_s_* (°C)	*A_f_* (°C)	*M_s_* (°C)	*M_f_* (°C)	∆*H_MA_* (J/g)	∆*H_AM_* (J/g)	*D_TH_* (°C)
As-Ti_50_Ni_50_	−46.7	26	−7.5	−71.8	16.3	−11.0	33.5
As-Ti_50_Ni_45_Cu_5_	25.4	66.1	36.8	8.3	28.9	−30.3	29.3
As-Ti_50_Ni_43_Cu_7_	0.7	32	13	−17.6	24.7	−25.8	19
HR-Ti_50_Ni_50_	51	68.4	36.3	15.9	3.8	−4.2	32.1
HR-Ti_50_Ni_45_Cu_5_	32.3	59.2	29.2	3.8	3.9	−4.4	30
HR-Ti_50_Ni_43_Cu_7_	32.2	54.3	32	12.6	3.5	−3.7	22.3

**Table 3:** Statistic values from the tensile stress–strain curves of samples in as cast and hot rolling conditions.

	Composition	First Plateau Stress (MPa)	Tensile Strength (MPa)	Strain (%)
	As-Ti_50_Ni_50_	93.4 (±10)	654.5 (±25)	21.7 (±5)
	As-Ti_50_Ni_45_Cu_5_	166.2 (±10)	565.2 (±25)	18.3 (±5)
	As-Ti_50_Ni_43_Cu_7_	294.9 (±10)	508.4 (±25)	14.4 (±5)
RD	HR-Ti_50_Ni_50_	186.2 (±5)	976.4 (±15)	29.2 (±2)
	HR-Ti_50_Ni_45_Cu_5_	58.8 (±5)	934.6 (±15)	44.9 (±2)
	HR-Ti_50_Ni_43_Cu_7_	39.3 (±5)	840.0 (±15)	32.4 (±2)
ND	HR-Ti_50_Ni_50_	185.8 (±5)	967.6 (±15)	28.3 (±2)
	HR-Ti_50_Ni_45_Cu_5_	59.0 (±5)	903.7 (±15)	38.2 (±2)
	HR-Ti_50_Ni_43_Cu_7_	39.1 (±5)	829.3 (±15)	30.6 (±2)

## Data Availability

The original contributions presented in this study are included in the article. Further inquiries can be directed to the corresponding author.
